# Developing Graphene Grids for Cryoelectron Microscopy

**DOI:** 10.3389/fmolb.2022.937253

**Published:** 2022-07-13

**Authors:** Hongcheng Fan, Fei Sun

**Affiliations:** ^1^ National Key Laboratory of Biomacromolecules, CAS Center for Excellence in Biomacromolecules, Institute of Biophysics, Chinese Academy of Sciences, Beijing, China; ^2^ University of Chinese Academy of Sciences, Beijing, China; ^3^ Center for Biological Imaging, Institute of Biophysics, Chinese Academy of Sciences, Beijing, China; ^4^ Bioland Laboratory, Guangzhou, China

**Keywords:** air–water interface, beam-induced motion, cryoelectron microscopy, graphene grids, grid production, preferred orientation

## Abstract

Cryogenic electron microscopy (cryo-EM) single particle analysis has become one of the major techniques used to study high-resolution 3D structures of biological macromolecules. Specimens are generally prepared in a thin layer of vitrified ice using a holey carbon grid. However, the sample quality using this type of grid is not always ideal for high-resolution imaging even when the specimens in the test tube behave ideally. Various problems occur during a vitrification procedure, including poor/nonuniform distribution of particles, preferred orientation of particles, specimen denaturation/degradation, high background from thick ice, and beam-induced motion, which have become important bottlenecks in high-resolution structural studies using cryo-EM in many projects. In recent years, grids with support films made of graphene and its derivatives have been developed to efficiently solve these problems. Here, the various advantages of graphene grids over conventional holey carbon film grids, functionalization of graphene support films, production methods of graphene grids, and origins of pristine graphene contamination are reviewed and discussed.

## Introduction

The successful application of direct electron detection devices (Ruskin et al., 2013; McMullan et al., 2014; Wu et al., 2016) and well-developed imaging processing algorithms (Scheres, 2012; Li et al., 2013a; Punjani et al., 2017; Zheng et al., 2017; Caesar et al., 2020; Punjani et al., 2020; Kimanius et al., 2021; Nakane and Scheres, 2021; Punjani and Fleet, 2021) have greatly improved the resolution of cryoelectron microscopy (cryo-EM), transforming this method into an important approach for determining the structures of biological macromolecules at near-atomic resolution. Compared to the X-ray diffraction technique, cryo-EM does not require crystals and only requires a small amount of specimen in its physiological solution. Therefore, cryo-EM has unique advantages and has been successfully applied to the study of the near-atomic resolution structures of challenging protein complexes with high flexibility (Yan et al., 2015; Plaschka et al., 2017; Ramirez et al., 2019) and small proteins (Khoshouei et al., 2017; Fan et al., 2019; Zhang et al., 2019; Han et al., 2020; Nygaard et al., 2020).

**FIGURE 1:**
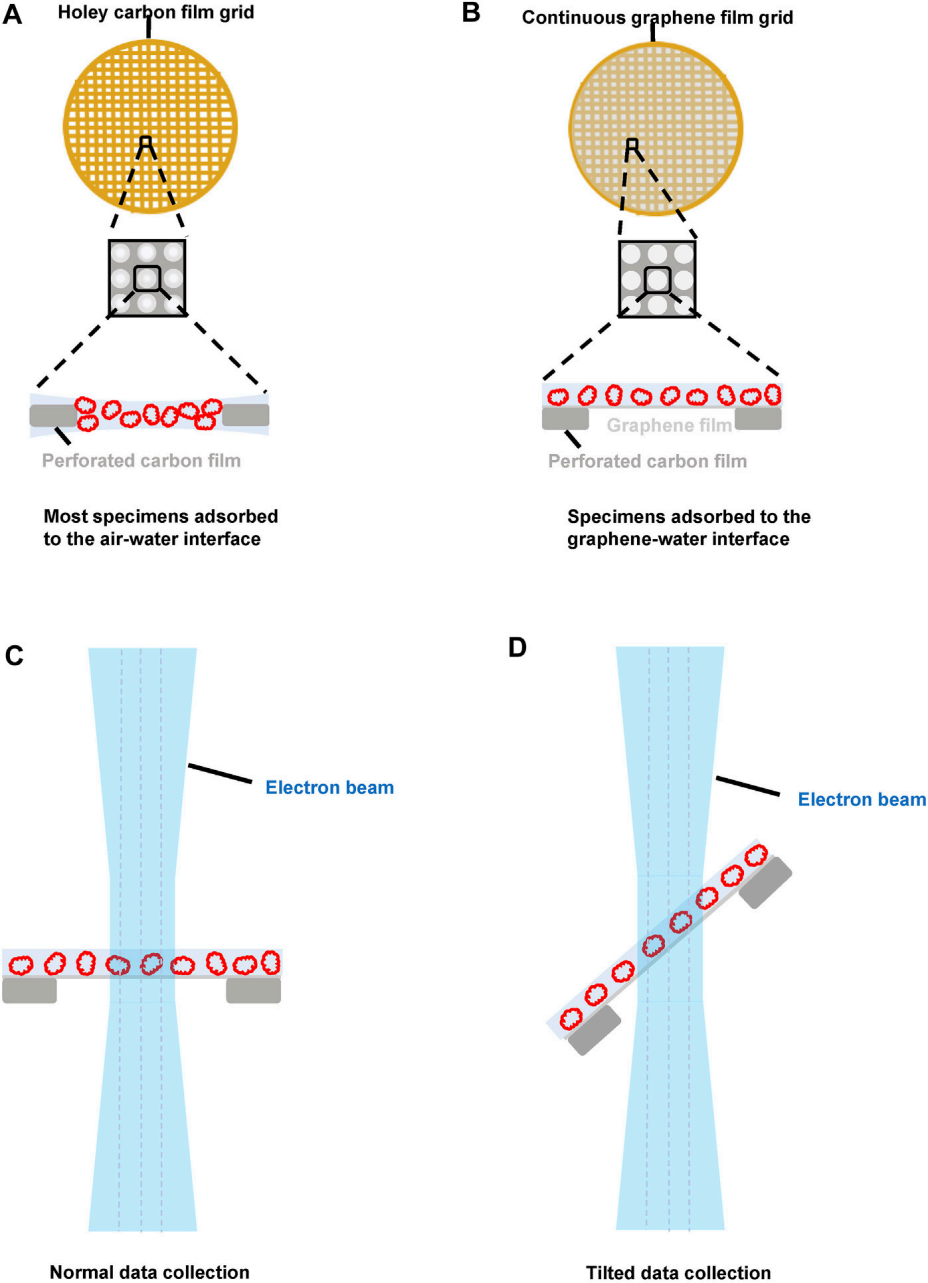
The potential advantages of pristine graphene grids in cryo-EM sample preparation. **(A)** the sample distribution using the holey carbon grid. Most protein particles are adsorbed onto the air–water interface. **(B)** the sample distribution using the graphene grid. Due to the interaction between the sample and the graphene–water interface, the protein particles can be kept away from the air–water interface. **(C)** normal single particle data collection using the graphene grid. The graphene grid can reduce the beam-induced motion. Thin and uniform ice using the graphene grid means that we can choose a smaller defocus without losing contrast. Most protein particles adsorbed onto the graphene layer are roughly in the same plane, which makes the subsequent contrast transfer function estimation more accurate. **(D)** application of the graphene grid for the tilt data collection strategy, which is a general solution to improve the map quality when the preferential orientation problem occurs (Lyumkis, 2019).

However, through cryo-EM studies, researchers have found that the conventional cryo-EM procedure does not always work and that there are obstacles in the specimen preparation procedure. The conventional plunge freezing procedure for cryo-EM sample preparation developed by Dr. Jacques Dubochet in the early 1980s (Dubochet et al., 1988) is still widely used. It comprises three steps. First, a drop of protein solution is applied to a holey carbon film grid that has been pretreated by plasma cleaning. Second, the excess solution is blotted using filter paper, resulting in a thin liquid film spanning across the grid holes. Third, the grid is rapidly plunged into a liquid cryogen, such as liquid ethane, which has been precooled using liquid nitrogen. After plunge freezing, protein particles are fixed in a thin vitrified ice layer (Figure 1A). With this procedure, the distribution of protein particles is not always ideally uniform in sufficiently thin ice, and many problems could be encountered in different projects, including a high noise background due to thick ice, a nonuniform distribution of particles within holes (Snijder et al., 2017; Drulyte et al., 2018), beam-induced motion (Glaeser, 2016), air–water interface–induced specimen denaturation/degradation (Glaeser et al., 2016), and preferred particle orientation (Tan et al., 2017). These problems have become bottlenecks for high-resolution cryo-EM studies in many cases.

In recent years, many efforts have been applied to developing various methods and techniques to solve the problems that can occur during cryo-EM specimen vitrification. One method is modifying the surface of the holey carbon support foil by manipulating glow discharging protocols (Isabell et al., 1999; Nguyen et al., 2015) or treatment with PEG (Meyerson et al., 2014) or detergents (Cheung et al., 2013). This type of approach can improve the particle distribution in the hole. A multiple blotting approach was proposed to increase the number of particles in the hole (Snijder et al., 2017). Holey metal support foils, including gold foil (Russo and Passmore, 2014b; Naydenova et al., 2020) and amorphous nickel–titanium alloy (ANTA) foil (Huang et al., 2021), were developed to decrease nonspecific interactions between particles and foils and reduce beam-induced motion. New types of cryo-EM sample preparation instruments have also been developed, such as Spotiton (Noble et al., 2018b), Vitrojet (Ravelli et al., 2020), and TED (Kontziampasis et al., 2019), to minimize the time of the vitrification procedure and to address the air–water interface problem.

Another type of technique is to coat the holey carbon/metal support foil of the grid with a continuous film, such as ultrathin amorphous carbon film (Grassucci et al., 2007), lipid monolayers (Kelly et al., 2008; Kelly et al., 2010), 2D crystals of hydrophobin HFBI (Fan et al., 2021), 2D crystals of streptavidin (Wang et al., 2008; Han et al., 2016; Han et al., 2017), and graphene material film (Wilson et al., 2009; Pantelic et al., 2010; Pantelic et al., 2011; Pantelic et al., 2012; Russo and Passmore, 2014a; van de Put et al., 2015; Palovcak et al., 2018; D'Imprima et al., 2019; Fan et al., 2019; Liu et al., 2019; Han et al., 2020; Wang et al., 2020a; Wang et al., 2020b; Liu et al., 2021). This method can improve the particle density in the hole and keep particles away from the air–water interface, with many successful examples. However, the type of amorphous carbon film normally used (approximately 2–5 nm) can add extra background noise and increase the difficulty of accurate image alignment. Thus, it is only suitable for large particles, such as ribosomes with molecular weights higher than 500 kDa (Thompson et al., 2016). The application of a streptavidin monolayer on the grid is sophisticated, and the additional lipid monolayer–coated ultrathin carbon film also generates a nonnegligible background. The performance of HFBI crystal films needs to be further investigated. Pristine graphene demonstrates superior qualities, including atomic-level thickness (3.4 Å), electron transparency (Pantelic et al., 2011), remarkable electrical conductivity (Geim and Novoselov, 2007), thermal conductivity (Balandin et al., 2008), and good mechanical strength (Lee et al., 2008). With these properties, pristine graphene has been proposed to be an ideal transmission electron microscopy (TEM) specimen support and has helped to overcome many challenges in sample preparation procedures (Meyer et al., 2008; Wilson et al., 2009; Pantelic et al., 2010; Pantelic et al., 2011; Pantelic et al., 2012; Russo and Passmore, 2014a; van de Put et al., 2015; Palovcak et al., 2018; D'Imprima et al., 2019; Fan et al., 2019; Liu et al., 2019; Han et al., 2020; Wang et al., 2020a; Wang et al., 2020b; Liu et al., 2021).

In this review, various advantages of graphene grids are discussed by comparing them with conventional holey carbon grids. In addition, to form an overall outline of the current development of graphene grids for cryo-EM, the recent progress in graphene film functionalization and the preparation of high-yield and clean graphene grids are discussed. If not specified, the phrase “graphene grid” represents both grids coated with graphene oxide film and grids coated with continuous pristine graphene film.

## Advantages of Graphene Grids

### A Brief Early History of Graphene Grid Development

The successful exfoliation of 2D crystal monolayer graphene films was realized by Andre Geim and Konstantin Novoselov in 2004 (Novoselov et al., 2004). Since its discovery, pristine monolayer graphene has been applied in numerous scientific fields because of its superior qualities, including atomic-level thickness (3.4 Å), remarkable electrical (Geim and Novoselov, 2007) and thermal conductivities (Balandin et al., 2008), optical properties (Ghuge et al., 2017), chemical inertness (Bellunato et al., 2016), and good mechanical strength (Lee et al., 2008). According to a review of earlier work on graphene grid development, the first work using a pristine graphene film as a TEM specimen support for imaging light atoms and molecules was performed in 2008 (Meyer et al., 2008). Afterward, graphene grids began to be applied in the biological TEM field, such as the imaging of positively stained DNA (Pantelic et al., 2011), vitrified influenza virus (Sader et al., 2013), and frozen-hydrated apoferritin (Sader et al., 2013).

However, unmodified graphene was not widely used, due to contaminant-induced hydrophobicity and degradation of image quality, until 2014, when Russo and Passmore et al. (2014a) adopted low-energy hydrogen–plasma treatment to render a pristine graphene film hydrophilic and found that the beam-induced motion could be efficiently decreased when using a graphene film–covered grid. Later, D’Imprima et al. (2019) proved that the denaturation effect of fatty acid synthase caused by the air–water interface (AWI) can be efficiently addressed using hydrophilized graphene–coated grids. Around the same time, Fan et al. (2019) developed their own high-yield and clean monolayer pristine graphene–coated grids, and they used these grids to determine the structure of streptavidin with a small molecular weight of 52 kDa to near-atomic resolution with cryo-EM.

Graphene oxide (GO) was also introduced as a substrate material for cryo-EM experiments since it is naturally hydrophilic, nearly electron transparent, and easy to synthesize (Wilson et al., 2009; Pantelic et al., 2010). However, it is not easy to prepare a grid uniformly covered with a monolayer or a few layers of GO because GO is normally fragmented and easily self-aggregates. To address this problem, a simple and robust method to make GO-coated grids was reported and used to determine the 2.5-Å cryo-EM structure of the 20-S proteasome (Palovcak et al., 2018). The GO-coated grids can also be used to determine the structures of small protein particles with molecular weights lower than 100 kDa with cryo-EM (Patel et al., 2021).

### Improved Sample Distribution and Thinner Uniform Ice

Vitrified particles in a hole may not look like what we expect. The ideal model of cryo-EM samples shows evenly distributed particles with random orientations, and the vitreous ice is uniformly thin. Owing to the interaction of particles with the air–water interface, support film, and neighboring particles, different situations can occur (Drulyte et al., 2018).

Next, the vitrified ice thickness in the hole affects the final resolution that we can achieve. Thick ice increases the background and therefore reduces the image contrast. In addition, the problems of defocus gradient (Zhang and Zhou, 2011; Sun, 2018) and high-frequency information dampening (Voortman et al., 2011) become severe when the thickness of ice increases. Therefore, the ice thickness needs to be optimized to just cover the size of the particle to minimize the background. However, many specimens, especially membrane proteins, preferentially remain within thick ice (Han et al., 2020; Yokoyama et al., 2020). In addition, when the vitrified ice layer becomes too thin, the particles can be pushed toward the edge of the support film, causing aggregations of particles. Furthermore, some protein particles become deformed or denatured by the surface tension at the air–water interface (Unwin, 2013; Cheng et al., 2015; D'Imprima et al., 2019; Yokoyama et al., 2020).

Thus, finding suitable areas that have both thin ice and a high density of evenly distributed particles in samples prepared on holey carbon grids remains challenging. Van de Put et al. (2015) achieved much thinner ice and uniform particle distribution by adsorbing specimens onto a GO support film. According to their results, an ultrathin vitrified ice layer with a thickness of 10 nm can be formed for high-resolution cryo-EM imaging of double-stranded DNA (300 bp), while the thickness of the ice layer is 130 nm using a conventional holey carbon grid. Han et al. (2020) determined a 2.6-Å resolution structure of streptavidin (52 kDa) using pristine graphene–coated grids. The ice thickness in their research was sufficiently thin, resulting in a very good contrast, even under a small defocus of −0.85 μm.

In most cases, protein particles are not at the same Z-height when they are vitrified using a conventional holey carbon film grid (Figure 1A), resulting in defocus variations for different particles. Although these variations can potentially be corrected during the image processing step using the contrast transfer function (CTF) refinement algorithm, using graphene grids, most protein particles are adsorbed onto the graphene support film and then kept roughly at the same Z-height (Figure 1B), which makes defocus estimation more accurate and reduces the computational cost of CTF refinement.

In some cases, when using a holey carbon film grid, the nonspecific interaction between protein particles and carbon film is nonnegligible, resulting in few particles found in the holes, while more particles are adsorbed on the carbon film. To obtain more particles in the holes, a high concentration of specimen would be necessary. However, using GO grids, Palovak et al. (2018) found that the concentration of 20-S proteasome could be 10 times lower than that using holey carbon film due to the interaction between the particles and the hydrophilic GO film. In addition, Han et al. (2020) reported that using pristine graphene grids, they observed a five times higher density of protein particles in the hole in comparison with that using a holey carbon film, and the particle distribution was more even. Therefore, graphene grids have the potential to yield a uniform distribution and high density of particles in the hole, which is expected to be important for studying membrane protein complexes reconstituted in liposomes (Yao et al., 2020).

### Protecting Specimens From the Air–Water Interface

Many cryo-EM research groups have found that the quality of vitrified samples deteriorates in comparison with that of negative-stained samples. This phenomenon was later explained by the interaction of protein particles with the air–water interface.

When the protein particles are confined to the thin layer of the solution on the grid, the particles can diffuse and approach the air–water interface quickly. It was estimated that there were more than 1,000 collisions per second with the air–water interface in the thin ice (≤100 nm), which gives sufficient opportunity for adsorption of particles in preferential orientation (Taylor and Glaeser, 2008). Using the Stokes–Einstein equation, we calculated that the average time of particles (10-nm diameter) approaching the air–water interface in thin ice (40-nm thickness) was approximately 6 ms (Sun, 2018). After approaching the air–water interface, the proteins may adopt a preferential orientation or desorb away from the air–water interface (Glaeser, 2018). Next, Noble et al. (2018a) found that approximately 90% of particles were adsorbed at the air–water interfaces with a preferred orientation. In addition, the forces at the air–water interface may cause different degrees of denaturation and dissociation of protein complexes (Glaeser, 2018). This denaturation effect occurs frequently during conventional cryo-EM sample preparation (Glaeser and Han, 2017).

To minimize the effect of the air–water interface, many approaches, including adding the surfactants OG (Benton et al., 2018), amphipol (Owji et al., 2020), CHAPSO (Chen et al., 2019), and fluorinated Fos-choline-8 (Popot, 2018; Wang et al., 2019), collecting data from the thicker ice regions, utilizing affinity grids (Lahiri et al., 2019), and coating the holey carbon grid with a continuous thin carbon film (Thompson et al., 2016), were attempted and were successful for certain specimens. However, these approaches introduce a significant additional image background, necessitate exhaustive trials without a clear sign of success, or induce a new preferred orientation. In addition, certain new vitrification instruments were invented to address the air–water interface problem (Razinkov et al., 2016; Arnold et al., 2017; Ravelli et al., 2020). For example, Spotiton can minimize the spot-to-plunge time to 100–200 ms, which can reduce the number of particles adsorbed at the air–water interface (Razinkov et al., 2016; Noble et al., 2018b; Darrow et al., 2019). Using a time-resolved cryovitrification device (Kontziampasis et al., 2019), Klebl et al. (2020) further reduced the time taken to vitrify particles that adsorb at the air–water interface within 6 ms, which improved the cryovitrification quality of some specimens (Klebl et al., 2020). However, these instruments are expensive, contain many special consumable materials, are not easily accessible by most research groups, and can only partially address the air–water interface problem.

In addition to our recent development of HFBI film–coated grids (Fan et al., 2021), the emergence of graphene grids provides a new solution to the air–water interface problem. With graphene grids, protein particles can adsorb at the graphene–water interface layer, thereby preventing protein particles from diffusing to the air–water interface (Figure 1B), which can address the issues of the air–water interface–induced preferred orientation, denaturation, and dissociation effects (D'Imprima et al., 2019; Fan et al., 2019; Naydenova et al., 2019; Han et al., 2020; Joppe et al., 2020). Compared to the approach of using a continuous thin carbon film, graphene grids induce less extra background and are applicable to small particles. However, a potential new preferential orientation problem can arise due to the interaction between the protein particles and the layer of the graphene–water interface. In addition, if the ice is too thin, the risk of exposing the protein particles to the air–water interface still exists.

### Reducing Beam-Induced Motion

When irradiated using an electron beam, the particles embedded in vitreous ice move, and this type of motion is called beam-induced movement (BIM). The BIM process involves two phases, including an initial rapid “burst” phase and a following slower phase (Glaeser, 2016). The burst phase may reflect the irradiation relieving stress that had been accumulated during plunge freezing, and the slower phase has three origins. The first origin is charging of the specimen, which has two subsequent effects, including electrostatic force–induced mechanical motion (Glaeser, 2016) and mini electrostatic lens–caused image deflection (Brink et al., 1998). A careful analysis has demonstrated that charging is not the dominant effect on image quality degradation (Russo and Henderson, 2018a; Russo and Henderson, 2018b). The second origin is radiation damage of proteins and amorphous ice, which can generate hydrogen gas, thereby causing a bubbling effect and introducing additional mechanical stress (McBride et al., 1986; Chen et al., 2008; Glaeser, 2008; Sun, 2018). The third origin is the beam-induced Brownian motion of water molecules (McMullan et al., 2015). The influence of this type of motion needs to be taken into account when the target resolution is smaller than 2 Å or a small particle size is studied (Sun, 2018).

The BIM effect can cause blurring of images and limit the achievable resolution of cryo-EM. Regarding obtaining a 3-Å resolution reconstruction, the elimination of the BIM effect can significantly reduce the number of particles needed to reach the same resolution by ∼30-fold (Henderson, 2018). Owing to their high frame rate and detective quantum efficiency, the emergence of direct electron detectors facilitated video recordings and the possibility of motion correction (Mooney et al., 2011; Campbell et al., 2012; Bai et al., 2013; Li et al., 2013a; Zheng et al., 2017; Zivanov et al., 2019); therefore, the largest motions in the slower phase can be corrected, which marked the beginning of the resolution revolution in cryo-EM (Kuhlbrandt, 2014). However, the motion in the rapid “burst” phase is erratic and cannot be effectively corrected by the motion correction approach, which means that the first few frames, with less radiation damage and containing high-resolution information, cannot be effectively utilized (Grant and Grigorieff, 2015).

The pristine graphene film–coated grids showed a unique advantage in reducing the BIM effect due to their high mechanical strength and electrical conductivity. Russo and Passmore (2014a) found that beam-induced motion could be reduced by a factor of ∼1.3 when adding a pristine graphene layer to a holey carbon grid (Figure 1C). This motion could be further decreased by a factor of ∼3 when a holey gold-foil–coated gold grid was covered with a pristine graphene film, taking advantage of the high electrical conductivity and mechanical stiffness of both the pristine graphene and gold films (Russo and Passmore, 2014b; Naydenova et al., 2019). In addition, the graphene lattice can be used as a fiducial to potentially improve the movie alignment (Palovcak et al., 2018).

We note that Naydenova et al. (2020) recently designed a new type of all-gold grid called HexAuFoil, which can decrease BIM to less than 1 Å by limiting the critical aspect ratio (hole diameter:ice thickness) to <11:1. However, this holey support cannot solve the air–water interface problem. The combination of the graphene film and this all-gold HexAuFoil support would be a new option to further improve the quality of cryo-EM sample preparation.

### Improving Particle Orientation Distribution

The preferred orientation of particles is another common limiting factor in cryo-EM single particle analysis and can be induced by interaction between the protein particles and the air–water interface, the support film, or the neighboring particles. A preferred orientation causes a biased distribution of angular projections and yields an anisotropic resolution in the final reconstruction (Tan et al., 2017); this is especially severe when the particles have low or no symmetry.

To address the issue of air–water interface–induced preferred orientation, in addition to the above approaches of minimizing the interaction between the particles and the air–water interface, methods for altering the properties of the supporting film or the air–water interface, including plasma treatment in the presence of N-amylamine (Miyazawa et al., 1999; da Fonseca and Morris, 2015; Nguyen et al., 2015), the addition of polylysine (Lander et al., 2012; Zang et al., 2016), and the use of self-assembled monolayers (Meyerson et al., 2014), have been tested on specific types of samples. As discussed above, the graphene grids can efficiently yield sufficiently thin ice; therefore, using graphene grids, more particles with different orientations can be selected, even for views with less contrast that are possibly ignored in holey carbon grid samples. In addition, graphene grids can efficiently keep particles away from the air–water interface and thus allow more orientations of particles.

To address the existence of a preferred orientation, Tan et al. (2017) developed a data collection strategy of tilting the specimen to remedy the anisotropic resolution problem, and they successfully applied this strategy to determine the high-resolution structures of the influenza hemagglutinin trimer and the large ribosomal subunit assembly intermediate. However, additional issues occurred for the dataset collected at the tilted angle, which included thicker ice, focus gradient, and increased specimen drift. These new problems, especially increased specimen drift, degrade both the quality of the micrographs and the speed of data collection, thereby limiting the potential of this data collection strategy for high-resolution structure determination.

These problems of the tilt data collection strategy can be mitigated using graphene grids. First, a much thinner and uniform vitrified ice can be prepared, thus minimizing the concerns about the increase in the thickness of the ice for tilted specimens. Second, since most protein particles are adsorbed onto the layer of the graphene–water interface and sitting on the same plane, the focus gradient can be more accurately estimated, and CTF estimation can be performed more precisely. Third, more importantly, with their high electric conductivity and mechanical stiffness, graphene grids can effectively reduce BIM even under the condition of specimen tilting. Hence, a tilt data collection strategy using graphene grids would be a better solution for coping with preferred orientation problems in high-resolution structural studies (Figure 1D); the work of Patel et al. (2021) showed that GO-coated gold foil grids could be used to collect tilted data of higher quality than that collected using a holey carbon grid.

In particular, graphene grids can prevent the contact of protein particles with the air–water interface but cannot solve the preferred orientation problem due to the interaction between the particles and the graphene–water interface. The tilt data collection strategy with graphene grids would be necessary for many specimens. Of late, we developed a new type of grid based on a 2D crystal HFBI film that can adsorb protein particles by means of electrostatic interaction to protect particles from the air–water interface and play a role similar to that of a graphene grid, with minimal background, and to help form thin enough ice (Fan et al., 2021).

## Functionalization of Graphene Grids

Monolayer graphene is an ideal support for cryo-EM studies in comparison with holey carbon films due to the above advantages. To address the new potential for preferred orientation from the interaction between protein particles and the graphene–water interface and to increase the affinity of the graphene support to the specific molecules, there are many developments involving functionalizing graphene to regulate the interaction between the support and the protein particles.

**FIGURE 2:**
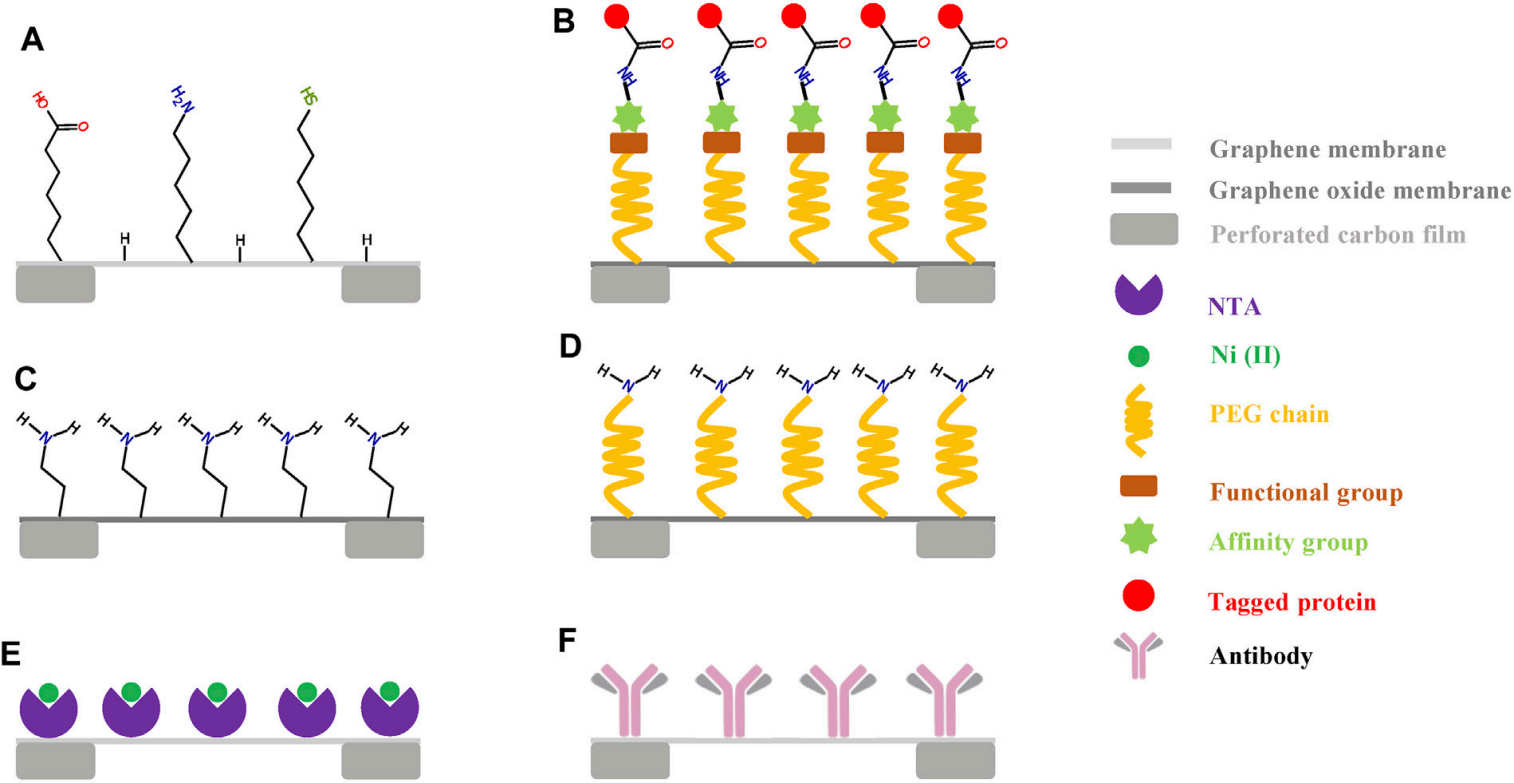
Functionalization of graphene grids. **(A)** the graphene film can be covalently functionalized with different organic molecules in a low-energy helium plasma (Naydenova et al., 2019). **(B)** the chemically functionalized graphene oxide (GO) grid has a special and general affinity for biomolecules fused with either SpyCatcher or SpyTag (Wang et al., 2019). **(C,D)** GO grid functionalized with amino groups or PEG-amino groups (Wang et al., 2020b). **(E)** the bioactive graphene grid can selectively capture His-tagged samples with the introduction of Ni–Nα,Nα-dicarboxymethyllysine groups onto the graphene surface (Liu et al., 2019). **(F)** the antibody-coated grid has a high and specific affinity for the target protein samples (Yu et al., 2016b).

The first approach is functionalizing a graphene film using plasma treatment (Figure 2A). Naydenova et al. (2019) covalently functionalized graphene films with different organic molecules in low-energy helium plasma. The amine covalently modified graphene grid could efficiently improve the orientation distributions of 30-S ribosomal subunits in comparison with those associated with the hydrogen-plasma treated graphene grid.

The second approach is to oxidize graphene to GO and then chemically modify GO (Figures 2B–E). Wang et al. (2019) established an affinity functionalization approach inspired by covalent bond formation between SpyCatcher and SpyTag (Figure 2B). They first anchored an amino-PEG-alkyne linker to the GO grid and then coupled an azide PEG spacer that was linked to SpyTag or SpyCatcher. Therefore, the chemically functionalized GO grid had a general affinity for biomolecules fused with either SpyCatcher or SpyTag. In addition, the presence of a flexible PEG spacer not only kept particles away from any surface (air–water interface and GO–water interface) but also allowed enough freedom to yield particles with different orientations. Wang et al. (2020b) also reported that the GO surface can be functionalized with amino groups or PEG-amino groups (Figures 2C,D). They found that the amino-GO and PEG-amino-GO grids showed better hydrophilicity, more protein adsorption, and better orientation distribution than the original GO grids. Liu et al. (2019) modified monolayer graphene by introducing Ni–Nα,Nα-dicarboxymethyllysine (Ni–NTA) onto the surface, and this functionalized graphene grid could specifically capture His-tagged proteins (Figure 2E), which can adsorb purified protein particles directly from cell lysates (Benjamin et al., 2016).

The third approach is to use reduced GO for high-resolution imaging (Liu et al., 2021). Compared to GO supports, reduced GO films were shown to have better electrical conductivity and a smaller interlayer space, which was proven to protect protein particles from the air–water interface and to facilitate the determination of the high-resolution structure of proteins with molecular weights smaller than 100 kDa (Liu et al., 2021).

The fourth approach is similar to a previously developed affinity grid used for cryo-SPIM (cryosolid phase immune electron microscopy) (Yu et al., 2016b). The affinity grid is made by immobilizing antibodies on the support film of the grid (Figure 2F) and has a high affinity for the target protein complexes based on the antigen–antibody interaction; this grid can be employed to adsorb purified protein particles directly from cell lysates. Yu et al. (2016b) demonstrated the feasibility of the affinity grid for studying various biological samples (including low abundance samples), whether purified or not. The affinity grid has also been successfully applied to study the morphology of pathogens such as human viruses (Lewis et al., 1988; Lewis, 1990; Lewis et al., 1995). The cryo-EM structure of Tulane virus with a low yield could be successfully determined to 2.6 Å using the affinity grid approach (Yu et al., 2016a). Considering the superior qualities of graphene supports (less background and high electrical conductivity) in comparison with that of carbon films, functionalizing graphene supports by immobilizing antibodies has great potential for wide application in studying the high-resolution structures of many challenging specimens.

## Production of Graphene Grids

With various advantages of graphene grids for cryo-EM study, many efforts to make reproducible and high-yield production of high-quality graphene grids have been performed in recent years. The nonuniform and low coverage as well as the surface contamination need to be efficiently solved during the production of graphene grids.

### Fabrication of Graphene Grids

Naturally hydrophilic GO is easy to obtain at low cost and has already been applied in cryo-EM studies (Pantelic et al., 2010; Palovcak et al., 2018; Patel et al., 2021). However, its propensity for fragmentation and self-aggregation tends to produce nonuniform, mutilayered coverage of the grid. In contrast, a large area of continuous monolayer pristine graphene can be grown on a metal substrate, such as a copper foil, by chemical vapor deposition (CVD) (Li et al., 2009a; Novoselov et al., 2012). However, the lack of a method for transferring monolayer pristine graphene to a grid with a high coverage rate, while avoiding contamination, is a major bottleneck in the preparation of high-quality graphene grids. Three types of methods have been developed to make graphene grids.

**FIGURE 3:**
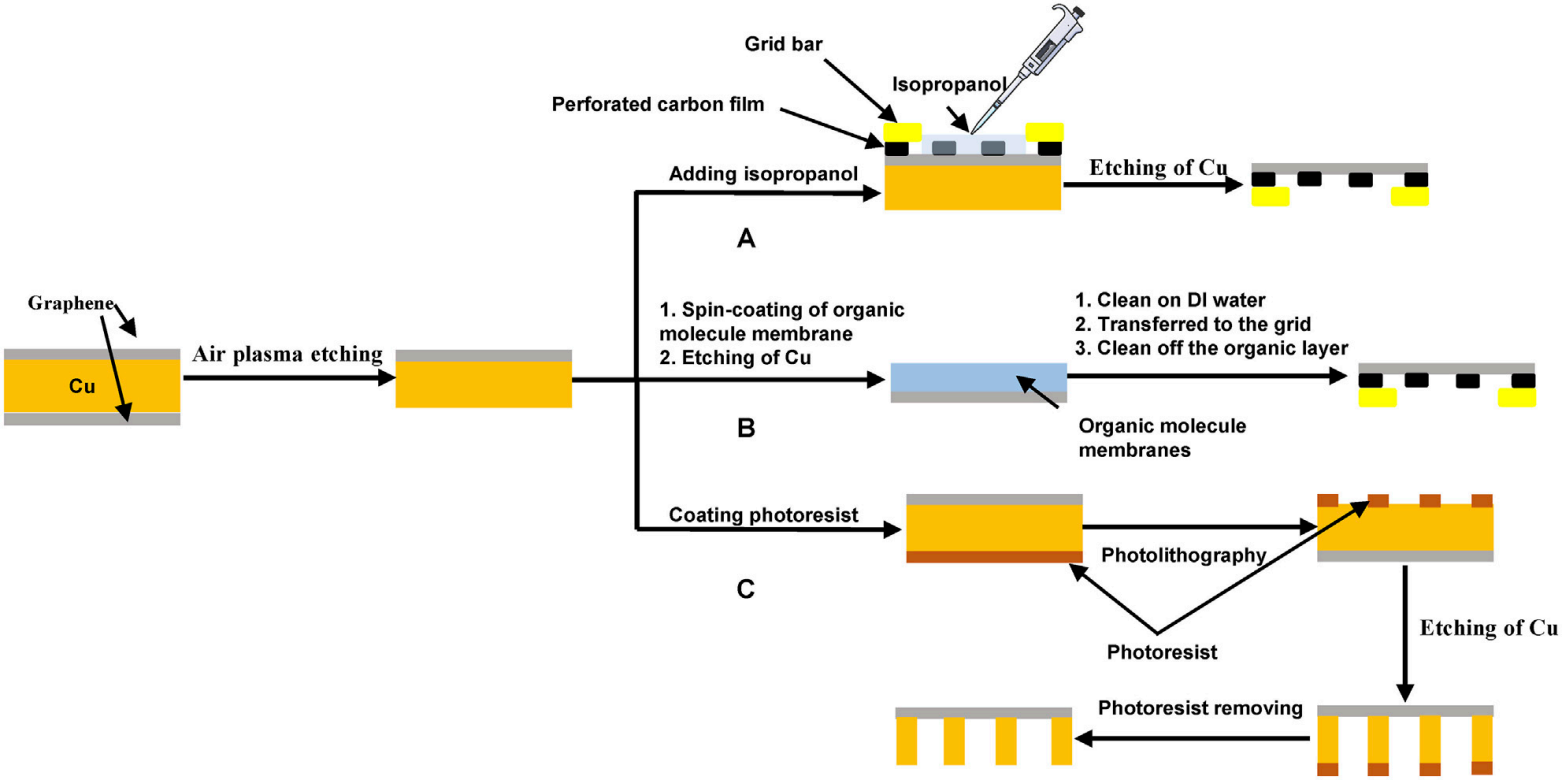
Fabrication of graphene grids. **(A)** a transfer-free method for preparing graphene grids. This method uses isopropanol to adhere a perforated carbon film onto a graphene film and then uses etchants, such as (NH_4_)_2_S_2_O_8_, Na_2_S_2_O_8_, and FeCl_3_ (Ullah et al., 2021) to etch away the copper substrate (Russo and Passmore, 2014a; de Martin Garrido et al., 2021). **(B)** organic molecule–assisted transfer method for preparing graphene grids. After the metal substrate, such as copper, is etched, different organic membranes, such as PMMA (D'Imprima et al., 2019), MMA (Han et al., 2020), colloid polymer (Naydenova et al., 2019), and paraffin (Leong et al., 2019; Qu et al., 2019), can be used to support the transfer of the graphene film to the grid and then can be dissolved using organic solvents, such as acetone. **(C)** direct etching method for fabricating graphene grids. The photoresist is used to make a pattern on the metal substrate, which is then selectively etched to complete the fabrication of graphene grids (Zheng et al., 2020).

The first method is transfer-free; a holey carbon grid is placed on the top of the graphene film, isopropanol is used to facilitate the adherence of the graphene film to the holey carbon foil by solvent wetting, and then an etchant, such as FeCl_3_, is used to remove the unwanted copper support of the graphene monolayer (Figure 3A) (Russo and Passmore, 2014a; de Martin Garrido et al., 2021).

The second method uses an organic layer to assist the transfer of the graphene film (Figure 3B and Table 1). D’Imprima et al. (2019) used polymethyl methacrylate (PMMA) to help transfer a graphene film to a grid. Han et al. (2020) chose a thin layer of methyl methacrylate (MMA) as the support during the transfer process. Warm acetone was used to dissolve and remove PMMA and MMA. PMMA contains carbonyl functional groups and has a strong noncovalent affinity with graphene (Leong et al., 2019), therefore resulting in a significant residue on the film. Compared to PMMA, less MMA was left on the graphene film after the transfer process due to its lower molecular weight. Naydenova et al. (2019) used collodion polymer to assist in the transfer of a graphene film onto a gold-foil-coated gold grid, where the collodion can be removed by dipping the grid into amyl acetate, 2-ethoxyethanol, chloroform, acetone, and isopropanol solvent in order. Compared to PMMA, the residual contamination, for example, nitrocellulose, could be circumvented by the combination of solvent cleaning and plasma treatment.

**TABLE 1:** Polymer-assisted transfer methods for fabricating graphene grids.

Polymer Support	Treatment	Advantages	Disadvantages	References
PMMA	Acetone solvent rinsing	Scalable, the most commonly used at present, high coverage rate	Polymer contamination, wrinkles and cracks	D'Imprima et al., 2019
MMA	Acetone solvent rinsing	Scalable, less residue contamination, high coverage rate	Wrinkles and cracks	Han et al., 2020
Collodion polymer	Amyl acetate, 2-ethoxyethanol, chloroform, acetone, and isopropanol solvent rinsing in order	Scalable, less residue contamination, high coverage rate	Wrinkles and cracks	Naydenova et al., 2019
Paraffin	Heating to 40°C, hexane solvent rinsing or removal by sublimination at 80°C	Scalable, less polymer contamination, wrinkles flattened; promising high coverage rate	The cryo-EM grid performance of this transfer method needs further validation	Leong et al. (2019), Qu et al. (2010)

Paraffin is a white or colorless, soft, solid wax made from saturated alkanes (Speight, 2020). Leong et al. (2019)developed an interesting approach that used a paraffin layer to achieve a residue-free and flattened transfer of a graphene film. Paraffin is adsorbed on the surface of graphene through noncovalent interactions and can be solubilized completely by organic solvents, such as hexane (Leong et al., 2019), or removed thermally (Qu et al., 2019). The difference in thermal expansion between graphene and its metal substrate is the source of the formation of graphene wrinkles (Deng and Berry, 2016; Wang et al., 2017), which can be efficiently avoided using paraffin-based transfer. Paraffin has a higher thermal expansion coefficient than PMMA (Ohashi and Paffenbarger, 1966). At the elevated temperature of 40°C, the paraffin film thermally expands and hence stretches the wrinkled graphene (Leong et al., 2019). Thus, in comparison with the PMMA-based transfer method, the paraffin-transferred graphene film is smooth and homogenous and shows enhanced electric conductivity closer to its intrinsic characteristic (Leong et al., 2019).

The third method is the use of direct etching to form the integral graphene grids without any transfer procedure (Figure 3C) (Aleman et al., 2010; Zheng et al., 2020). A large sheet of graphene was grown on a copper substrate using the CVD method, and the backside of the copper substrate was selectively etched using a photoresist-assisted method to make the copper foil. As a result, ultraclean graphene grids were prepared. Aleman et al. (2010) developed this method, but they exhibited problems with amorphous carbon and iron oxide contamination. Zheng et al. (2020) adopted this method to remove amorphous carbon contamination, and they chose Na_2_S_2_O_8_ as the etchant to dissolve unnecessary parts and then prepared cleaner graphene grids.

### Contamination and Cleaning

During the process of graphene grid fabrication, the graphene surface is exposed to contamination of different origins, such as airborne contamination (Li et al., 2013b), CVD-induced contamination (Lin et al., 2019), and polymer residual–induced contamination (Lin et al., 2012; Zhang et al., 2017; Leong et al., 2019; Schweizer et al., 2020).

Clean graphene is intrinsically hydrophilic and can form a strong H–π interaction with water molecules (Voloshina et al., 2011; Hamada, 2012). However, the graphene surface can easily adsorb hydrocarbons from ambient conditions in a short period of time. Different kinds and concentrations of hydrocarbons (alkanes, alkenes, alcohols, and aromatics) exist in the air (Millet et al., 2005). Schweizer et al. (2020) found that a thin and continuous recontamination layer appeared on freshly cleaned graphene after it was exposed to air for 5 min. The wettability of graphene can be reflected using the water contact angle (WCA). The WCA of monolayer graphene grown on a copper substrate using CVD and transferred to a SiO_2_ substrate was reported to be 90.4° (Kim et al., 2011), which is similar to the WCA (84°–86°) of graphite (Morcos, 1970; Werder et al., 2008). Li et al. (2013b) reported that as-prepared graphene grown on a copper substrate was surprisingly hydrophilic, with a WCA of 44°. When the graphene was exposed to air, its WCA quickly increased to 60° within 20 min and plateaued at 80° after 1 day. They concluded that hydrophobic molecules from the air could accumulate on the surface of graphene, resulting in a change in the WCA and making graphene hydrophobic.

These airborne contaminants could be partially removed using thermal annealing, plasma treatment, and ultraviolet-O_3_ treatment. For thermal annealing, exposure to an elevated temperature of ∼550°C for a relatively long time was needed to reduce the hydrocarbon contaminants (Li et al., 2013b); these contaminants would damage the graphene by introducing defects (Cancado et al., 2011). Plasma treatment is commonly used to increase the hydrophilicity of TEM grids (Isabell et al., 1999). However, conventional plasma cleaning using air, oxygen, or argon would quickly destroy the monolayer graphene within seconds. Russo and Passmore et al. (2014a) developed a low-energy hydrogen–plasma treatment to make a graphene monolayer hydrophilic without significant damage. Ultraviolet-O_3_ treatment can also effectively remove airborne contaminants at a slow and controllable rate using ozone gas oxidation (Han et al., 2020). To increase the hydrophilicity of graphene grids, D’Imprima et al. (2019) developed a noncovalent chemical doping method by coating the graphene surface with the compound 1-pyrene carboxylic acid (1-pyrCA) via π–π interactions. Their method preserved the pristine graphene structure without removing the hydrocarbon contaminants. In addition, when the concentration of 1-pyrCA was high, an extra background was introduced.

Lin et al. (2019) found that another type of amorphous carbon contamination could be introduced during CVD growth, called CVD-induced contamination. Cu is the catalyst that decomposes hydrocarbons (carbon precursors), forming sp^2^ crystalline carbon. With increasing graphene coverage, the graphitization process slows, and the formation of amorphous carbon becomes preferential (Robertson, 1996; Li et al., 2009b). Therefore, sufficient Cu catalytic activity is important during CVD growth. Cu foam mediation, owing to its high specific area, can furnish enough Cu vapors to decompose hydrocarbons and consequently restrain the formation of amorphous carbon (Lin et al., 2019). When the formation of sp3 amorphous carbon is suppressed, clean graphene can be obtained. This contamination can also be removed by posttreatment with CO_2_ developed by Zheng et al. (2020).

Since the monolayer graphene is very thin (3.4 Å), an extra support layer is needed when the graphene surface is isolated from the metal substrate. The PMMA layer is still the most widely used material for graphene transfer (Gao et al., 2021). However, polymer contamination is a major problem affecting the intrinsic properties of graphene. Lin et al. (2012) developed a thermal annealing method to remove PMMA contamination with two annealing steps. First, the layer of PMMA-A (PMMA facing the air) is decomposed at ∼160°C, and then, the layer of PMMA-G (PMMA facing the graphene) is removed at ∼200°C. Although annealing is an easy method for removing polymer contamination, there is still extensive PMMA residue on the graphene surface, even when annealing up to 700°C is performed, with the risk of graphene breakage. More importantly, the decomposition of PMMA is a kind of complex radical chain reaction. The radicals generated during annealing might covalently interact with the graphene defects, making PMMA residuals harder to remove. Organic solvents, such as acetone, do not work in this situation. In the future, searching for new organic molecules that do not require postannealing to decompose and are easily removed using organic solvents for use in the polymer-assisted transfer method is important.

## Concluding Remarks

The structure of apoferritin has been resolved at atomic resolution with the development of new hardware, including cold field emission guns, monochromators, aberration correctors, and the latest generation camera coupled to a new energy filter (Nakane et al., 2020; Yip et al., 2020), which indicates a new era of cryo-EM. However, sample preparation remains the major challenge for high-resolution structural determination using cryo-EM. Although holey carbon grids are still commonly used, developing other types of better grids to solve the problems of poor particle distribution, preferred orientation, air–water interface, beam-induced motion, etc., has been increasingly important to making cryo-EM more successful and efficient.

Monolayer graphene grids, with minimal background, have become a promising approach to solve these problems and offer the opportunity to reveal near-atomic structures of proteins with a small molecular weight (<100 kDa), low concentration, and even a transient intermediate state. For the samples that can be solved to a medium-high resolution using a holey carbon grid, the use of a graphene grid can significantly reduce the beam-induced motion, prepare uniform and thinner ice, and thereby increase the possibility of higher resolution.

As of now, most graphene grids for cryo-EM studies are prepared by researchers themselves and thus often are associated with poor reproducibility, a lower coverage rate, cleanliness problems, etc. With the development of more scalable and robust methods for fabricating high-quality and ultraclean graphene grids, further commercialization will be possible, and thus, monolayer graphene grids as well as different functionalization treatments will become widely used in the cryo-EM community and make cryo-EM sample preparation more successful and reproducible.
